# Early feeding of fortified breast milk and in-hospital-growth in very premature infants: a retrospective cohort analysis

**DOI:** 10.1186/1471-2431-13-178

**Published:** 2013-11-04

**Authors:** Christoph Maas, Cornelia Wiechers, Wolfgang Bernhard, Christian F Poets, Axel R Franz

**Affiliations:** 1 Department of Neonatology, University Children’s Hospital Tübingen, Calwerstr. 7, Tuebingen 72076, Germany

**Keywords:** Early neonatal growth, Enteral feeding, Infant premature, Human milk

## Abstract

**Background:**

Fortified human milk may not meet all nutritional needs of very preterm infants. Early transition from complementary parenteral nutrition to full enteral feeds might further impair in-hospital growth. We aimed to investigate the impact of the cumulative intake of fortified human milk on early postnatal growth in a cohort of very low birth weight infants after early transition to full enteral feeds.

**Methods:**

Retrospective single-centre observational study. Data are presented as median (interquartile range).

**Results:**

N = 206 very preterm infants were analysed (gestational age at birth 27.6 (25.6-29.6) weeks, birth weight 915 (668-1170) g). Full enteral feeds were established at postnatal day 8 (6-10) and adequate postnatal growth was achieved (difference in standard deviation score for weight from birth to discharge -0.105(-0.603 - -0.323)). Standard deviation score for weight from birth to day 28 decreased more in infants with a cumulative human milk intake >75% of all enteral feeds (-0.64(-1.08 - -0.34)) compared to those with <25% human milk intake (-0.41(-0.7 - -0.17); p = 0.017). At discharge, a trend towards poorer weight gain with higher proportions of human milk intake persisted. In contrast, we observed no significant difference for head circumference growth.

**Conclusions:**

Our current standardized fortification of human milk may not adequately support early postnatal growth.

## Background

Human milk feeding reduces the risk of necrotizing enterocolitis [1,2] and is associated with improved long-term outcome in very preterm infants [3,4]. On the other hand, several reports show an association of maternal milk feeding with early postnatal growth restriction in very preterm infants even if human milk was fortified [5,6]. This is most probably caused by intra- and interindividual variability of human milk composition resulting in deficits in macro- and micronutrient supply in some infants [7]. These negative effects on growth may be particularly relevant to very preterm infants receiving expressed breast milk early on.

We consequently investigated the relationship between the proportion of cumulative total enteral feeding volume administered as breast milk and early postnatal growth in a cohort of very preterm infants after early transition to full enteral feeds.

## Methods

This retrospective, non-consecutive three year cohort analysis was performed at Tübingen University Children’s Hospital. The ethics committee at the University of Tübingen, Faculty of Medicine, approved this retrospective evaluation and waived the need for parental consent, hence parental consent was not asked for.

### Study population

We evaluated all inborn infants with a gestational age (GA) <32 weeks at birth and a birth weight (BW) <1500 g, born in 2006, 2007 and 2010. The initial study [8] aimed at evaluating the effect of accelerated enteral feeding advancement on the time to full enteral feeds. As there was a transitional period (2008/09) after implementation of the new feeding guidelines, data was collected for two cohorts: infants born in 2006/07 and in 2010.

### Data collection

Exact nutritional intakes and anthropometric data were determined daily by detailed chart review for the first 28 days of life, then weekly, until discharge.

### Nutrition policy

A standardized feeding protocol was applied that defined feeding increments, handling of feeding difficulties, and complementary parenteral nutrition. Feeding of expressed breast milk of the infant's own mother was encouraged. Because donor milk was not available, supplemented breast milk was complemented with preterm formula (Beba preterm formula, Nestlé) if necessary to meet the prescribed enteral feeding volume.

The feeding policy in 2006/07 was to start enteral feeds on the first day of life with 10-15 ml/kg/d of preterm formula (Beba preterm formula, Nestlé). As soon as possible, preterm formula was replaced by breast milk. Daily feeding advancements were scheduled at increments of 15-20 ml/kg/d. Supplementation with a multicomponent fortifier (FM 85®, Nestlé; 1.0-1.5 g protein and 18-27 kcal per 100 ml) was started when enteral feeds reached 150 ml/kg/d.

In contrast, in 2010, enteral feeds were initiated with 20 ml/kg/d and advanced by 25-30 ml/kg/d. Breast milk fortification was started at a feeding volume of 100 ml/kg/d.

In cases of fluid restriction (total fluid intake < 150 ml/kg/d), the dosage of the multicomponent fortifier was increased up to 7.5% (equal to 1.5 g protein and 27 kcal per 100 ml) in both periods. At discretion of the attending neonatologist, the dosage of multicomponent fortifier was also augmented in infants showing persistently faltering growth.

### Assumptions and definitions

For calculation of macronutrient supply, we assumed a protein and energy content of 1.4 g/100 ml and 67 kcal/100 ml in human milk.

Full enteral feeding was defined as ≥140 ml/kg/day of milk feeds actually administered for more than 24 h.

Further details of the study population, exclusion criteria, nutrition policy and macronutrient supply have been reported previously [8].

### Measures of growth

From birth to discharge, weight was measured daily with electronic scales and frontooccipital head circumference (HC) weekly with a measuring tape.

Standard deviation scores (SDS) for weight and HC were computed using LMSgrowth (version 2.14; http://www.healthforallchildren.com/?product=lmsgrowth). The reference population was the British 1990 growth reference [9,10] fitted by maximum penalized likelihood as described before [10]. To account for the impact of intrauterine growth restriction, SDS-differences (SDS_discharge_ – SDS_birth_) and (SDS_d28_ - SDS_birth_) were calculated to illustrate in-hospital postnatal growth.

Data on linear growth were not reported due to the poor reliability of length measurements in the routine neonatal intensive care.

### Statistical analyses

Data are presented as median (interquartile range). Comparisons between cohorts were performed using the Wilcoxon/Kruskal-Wallis test or Fisher’s exact test. Statistical significance was assumed at p < 0.05.

Analyses were performed with JMP® 10.0.0 (SAS Institute Inc., USA).

## Results

206 of 240 inborn infants with a GA <32 weeks and a BW <1500 g had complete data sets and were analysed. GA at birth was 27.6 (25.6-29.6) weeks and BW 915 (668-1170) g. Full enteral feeds were established at postnatal d8 (6-10). A total of 197/206 infants (96%) received at least some breast milk. The proportion of cumulative total enteral feeding volume provided as breast milk was 86% (41%-95%) at d28 and 81% (33%-94%) at discharge.

**Figure 1:**
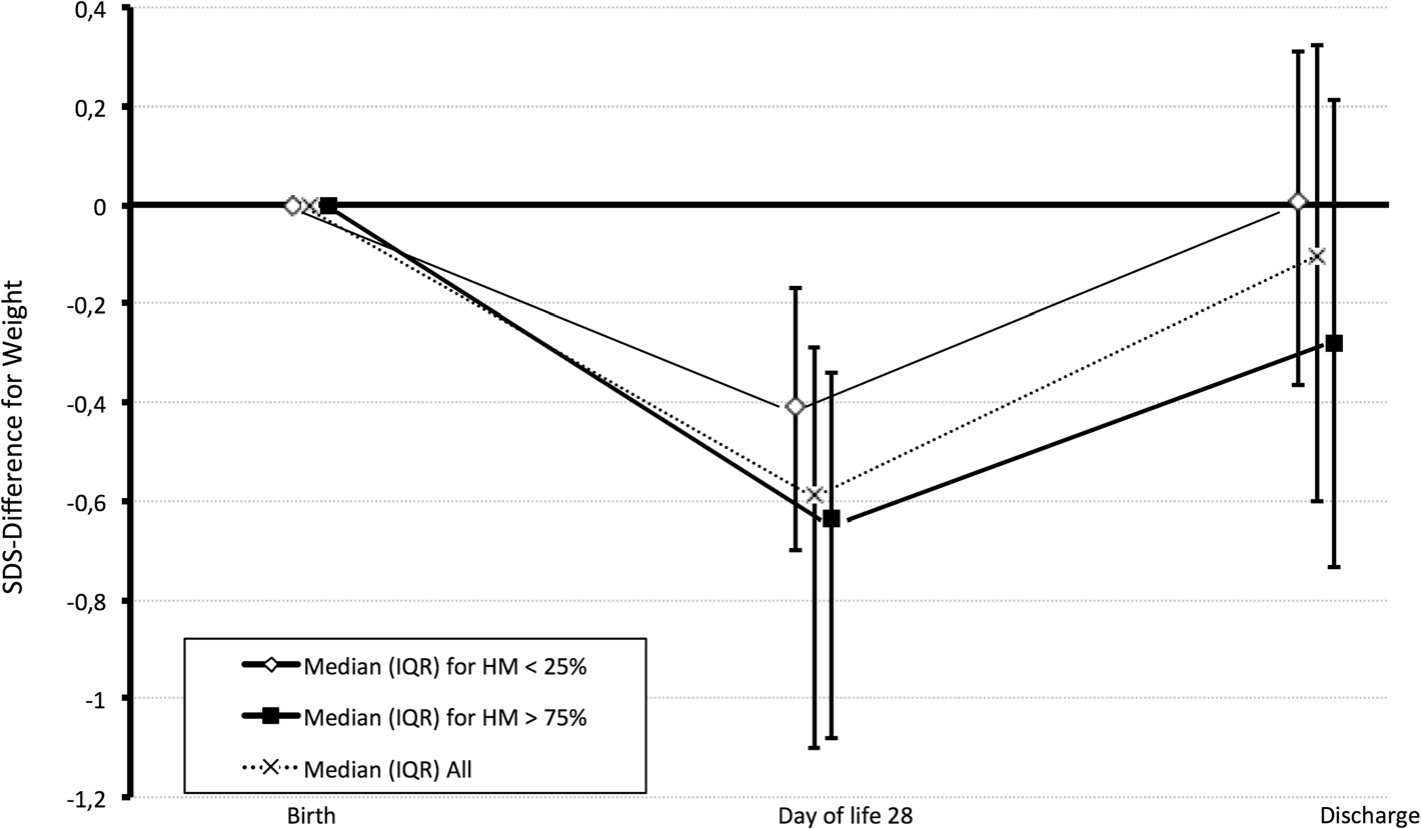
**Change in SDS-differences for weight during hospitalisation (median and interquartile range).** Box and whiskers is point estimate and interquartile range; horizontal line cutting y-axis in zero representing no differences in SDS for weight.

122/206 (d28) and 112/206 infants (discharge) received >75% human milk, whereas the proportion of human milk was <25% in 37/206 (d28) and 40/206 (discharge) infants, respectively. SDS-difference for weight from birth to d28 was significantly more negative with >75% cumulative human milk intake in comparison to the group receiving <25% human milk (Figure 1, p = 0.017). Comparing infants with >75% cumulative human milk intake versus those with <25% human milk intake, GA, BW, SDS for weight at birth (-0.995(-1.64- -0.27) vs. -1.28(-2.11- -0.64), p = 0.17), proportion of infants with SDS for weight at birth < -2 (9/37 vs. 25/122, p = 0.65), gender distribution, Clinical Risk Index for Babies (CRIB) and cumulative energy intake were similar (see Table 1). There was, however, a slightly lower calculated cumulative protein intake until d28 in the group receiving >75% human milk (3.86 (3.67-4.02) vs. 3.98 (3.77-4.26) g/kg/d; p = 0.023). The incidence of necrotizing enterocolitis was 3.6% in the group receiving > 75% human milk and 5% in the group receiving < 25% human milk (difference not significant, p = 0.33).

**Table 1:** Patient characteristics and outcome variables in relation to cumulative human milk intake until d 28

	**> 75% human milk**	**< 25% human milk**	**p-value by Wilcoxon test**
Number of infants [n/N] (m/f) [n/n]	122/206 (48/74)	37/206 (21/16)	
Gestational age at birth [weeks] median (IQR) (range)	27.6 (25.5-29.7) (23.0-31.7)	28.6 (25.6-30.5) (23.7-31.9)	p = 0.3
Birth weight [g] median (IQR) (range)	925 (665-1175) (290-1490)	846 (705-1160) (340-1490)	p = 0.9
CRIB-score median (IQR)	4 (1-7)	4 (1-6)	p = 0.42
Incidence of necrotizing enterocolitis [n/N]	3/122	2/37	p = 0.33 (Fisher’s exact)
Day of life when full enteral feeds were attained median (IQR)	8 (6-10)	8 (6-10)	p = 0.68
Cumulative energy intake (*) [kcal/kg] first four weeks of life median (IQR)	3431 (3219-3601)	3438 (3356-3562)	p = 0.58
Cumulative protein intake (**) [g/kg] first four weeks of life median (IQR)	105 (101-110)	109 (105-116)	p = 0.002
SDS-difference for weight (SDSd28 – SDSbirth) median (IQR)	-0.64 (-1.08-0.34)	-0.41 (-0.7-0.17)	p = 0.017
Postmenstrual age at discharge [weeks] median (IQR)	38.1 (36.5-40)	38.3 (36-40.3)	p = 0.48
Age at discharge [days] median (IQR)	72 (48-98)	69 (44-93)	p = 0.41

IQR = interquartile range.

(*) = assuming human milk energy content of 67 kcal/100 ml.

(**) = assuming human milk protein content of 1.4 g/100 ml.

SDS-difference for weight persistently tended to be lower until discharge with >75% vs. <25% cumulative human milk intake (SDS-difference for weight -0.28(-0.74- +0.21) vs. +0.01(-0.37- +0.31); p = 0.07). SDS-differences for weight during hospitalisation for all study infants and the two subgroups are displayed in Figure 1.

SDS-differences for HC were similar, both at d28 (-0.75(-1.48 - +0.06) vs. -0.58(-1.31 - +0.26); p = 0.2) and at discharge (0.1(-0.56- +0.76) vs. 0.52(-0.77 - +1.14); p = 0.24).

## Discussion

Following early transition to full enteral feeds with predominantly fortified human milk, we observed a significant drop in SDS for weight and a non-significant trend towards lower HC during the first four postnatal weeks, with the majority of infants returning to their growth trajectories until discharge (Figure 1). The latter was true most notably for infants receiving a cumulative human milk intake <25%. During the first four weeks of life a cumulative human milk intake >75% was associated with a significantly more severe decline in SDS for weight compared to children receiving <25% human milk (Figure 1). This difference persisted as a trend until discharge. These results are in line with previous reports [5,6] yet with remarkably better overall postnatal weight gain (median SDS-difference for weight of -0.28 at discharge with >75% human milk in this study, compared with -0.5 in the study by Colaizy et al. [6]).

In contrast to the observed differences in weight gain, no significant difference was observed in HC growth with different proportions of cumulative human milk intake, both, at d 28 and at discharge. Better HC growth than overall weight gain in predominantly human milk fed preterm infants is consistent with the previously delineated “breastfeeding paradox” in very preterm infants describing better neurodevelopmental outcome in spite of suboptimal initial weight gain [4].

Most likely the differences in weight gain can be attributed to intra- and inter-individual variability of human milk composition, particularly the early decline in protein content of human milk during lactation [11], resulting in insufficient protein supply in some infants predominantly receiving human milk if current standardized fortification is applied [7]. The variability in nutrient content of human milk is reflected in the wider (interquartile) ranges for SDS-difference for weight at d28 and discharge observed in infants receiving >75% human milk compared with those receiving <25% (Figure 1).

Future studies are required to show whether this potential protein deficit is best prevented by standardized supplementation with more protein given to all infants fed human milk, or via individual fortification of human milk after milk analysis.

Furthermore, optimization of the micronutrient content of human milk and formula to the needs of very preterm infants may be required for further improvement of growth [12].

Strengths of this study include the meticulous documentation of exact nutrient intakes along with anthropometric data expressed as SDS-changes during hospitalisation in early enterally fed very preterm infants. Additionally, the cohort included a high proportion of extremely immature infants who are at the highest risk of faltering postnatal growth. Limitations consist in the retrospective, observational, and single-centre design of the study. The fact that we do not report linear growth data because of potential poor reliability and that non-consecutive years were evaluated may also be perceived as limitation.

## Conclusions

Although adequate early postnatal growth can be achieved in early enterally fed very preterm infants, our current standardized fortification of human milk does not meet the needs of all infants. To prevent the observed small yet persisting growth deficit in predominantly human milk fed very preterm infants, special attention to intra- and inter-individual variability of protein content in human milk may be required.

## Abbreviations

SDS: Standard deviation score; GA: Gestational age; BW: Birth weight; HC: Head circumference.

## Competing interests

The authors declare that they have no competing interests.

## Authors’ contributions

CM designed the study, performed the analyses, drafted the initial manuscript and revised the manuscript; CW reviewed and revised the manuscript making important intellectual contributions; WB reviewed and revised the manuscript making important intellectual contributions; CFP supervised the project as the head of department and reviewed and revised the manuscript making important intellectual contributions; ARF was co-coordinator of the project, supervised data analyses and reviewed and revised the manuscript making important intellectual contributions. All authors read and approved the final manuscript.

## Pre-publication history

The pre-publication history for this paper can be accessed here:

http://www.biomedcentral.com/1471-2431/13/178/prepub
